# Weak Eyes

**Published:** 1878-12

**Authors:** 


### *WEAK EYES.*

The causes that induce weak eyes are many, as colds, catarrh, headache, scrofula, etc. The weakness is frequently very persistent in spite of remedies, and may last for years. The best remedies are perfect cleanliness, bathing the eyes frequently with warm water, and a proper attention to diet and to the condition of the general health. It is a mistake to favor the eyes too much by excluding the light and air. Such a course long persisted in would destroy the best and strongest eyes. It is very trying to the eyes to sit in a room that is dimly lighted, for they are involuntarily strained in the effort to see objects that are hardly visible. The entire exclusion of light for a few weeks produces complete blindness. The better plan is to have strong lights, softened by white, translucent shades.

